# Effect of previous *Chlamydia Trachomatis* infection on the outcomes of ivf/icsi treatment: a retrospective study

**DOI:** 10.1186/s12884-022-04624-z

**Published:** 2022-04-10

**Authors:** Dan Zhang, Zengyan Wang, Xingyuan Hu, Chunguang Ma, Yuanlin Ma, Yanwen Xu

**Affiliations:** 1 Center for Reproductive Medicine and Department of Obstetrics & Gynecology, The First Affiliated Hospital of Sun Yat-sen University, 58 Zhongshan Road II, Guangzhou, Guangdong 510080 People’s Republic of China; 2 Guangdong Province Key Laboratory of Reproductive Medicine, Guangzhou, People’s Republic of China; 3 Zhong-shan School of Medicine, Sun Yat-sen University, Guangzhou, People’s Republic of China; 4 Department of Dermatology, The First Affiliated Hospital of Sun Yat-sen University, Guangzhou, People’s Republic of China

**Keywords:** *Chlamydia trachomatis* infection, In vitro fertilization, Intracytoplasmic sperm injection, Pregnancy outcome

## Abstract

**Background:**

The impact of *Chlamydia trachomatis* infection (CT) on the outcomes of In-Vitro Fertilization / Intracytoplasmic sperm injection (IVF/ICSI) has been controversial.

**Methods:**

A total of 431 infertility women aged 20–38 years with or without *Chlamydia trachomatis* infection before fresh/ frozen embryo transfer were included to investigate the effect of cured CT infection. The infected group was divided into two subgroups for ≤3 months and > 3 months according to the different intervals between *Chlamydia trachomatis* positive testing and embryo transfer. The effect of chlamydia infection and the intervals between infection and embryo transfer on pregnancy outcomes was analyzed with correction for potential confounders within a multivariable model.

**Results:**

Our results revealed that implantation rate was significantly lower and the premature rupture of membranes (PROM) was higher in women with CT infection than non-infection. The multivariate logistic regression analysis adjusting for baseline characteristics showed no significant difference in live birth rate between neither two groups nor two subgroups.

**Conclusions:**

The study suggests that previous *Chlamydia trachomatis* infection would lead to high risk of PROM. The intervals between infection and embryo transfer would not impact the pregnancy outcomes of IVF/ICSI.

## Background

Genital *chlamydia trachomatis* (CT) infection is one of the most common sexually transmitted diseases throughout the world. Presenting as asymptomatic in most cases [1], chlamydial infections can cause infection for more than 1 year among up to 50% of the infected people if not treated in time [2], which can lead to a series of serious complications such as pelvic inflammatory disease [3], ectopic pregnancy, fecundity decline and even tubal obstruction [4, 5].

Since the infection of CT has shown adverse impact on human reproductive capability, however, the impact of CT infection on IVF/ICSI among infertile patients has been controversial and previous studies have reached different conclusions [6–9]. The presence of anti-Chlamydia antibodies or heat shock protein 60 (HSP 60) may contribute to autoimmune response and the subclinical infection, leading to poor pregnancy outcomes in IVF/ICSI cycles [8–10]. Some studies found that there was no significant difference in outcomes of IVF/ICSI between patients with and without CT infection [11–14].

Furthermore, there were few reports on the optimal time for embryo transfer after cure of chlamydia infection. As a result, we conducted a retrospective cohort study to further explore the impact of CT infection and the intervals between infection and embryo transfer on IVF / ICSI outcomes.

## Methods

### Study population

In this retrospective study, all women had screened for *Mycoplasma genitalium*, Chlamydia, bacterial vaginosis and trichomonas vaginalis before entering IVF/ICSI. Women who tested CT positive before oocyte retrieval or frozen embryo transfer of IVF/ICSI cycles were included in the experimental group when the CT test turned negative after one standard treatment. Women who tested CT negative without female tubal factors were enrolled as the control group from June 2018 to January 2020 of the First Affiliated Hospital of Sun Yat-sen University. The other inclusive criteria: (1) undergoing an IVF / ICSI program. (2) Age between 20 and 38 years. (3) BMI ≤ 28 kg/m2. (4) Normal ovarian reserve (bFSH < 10 IU/L). (5) Number of oocytes retrieved≥5. (6) Total number of oocytes retrieval cycles ≤3. (7) Endometrial thickness on hCG day or progesterone conversion day ≥7 mm. The exclusion criteria: (1) Patients with intrauterine abnormalities, including: endometrial polyps or submucosal fibroids, history of uterine adhesiolysis, uterine malformation. (2) Patients with a history of recurrent spontaneous abortion (RSA), RSA was defined as three or more consecutive pregnancies loss (including biochemical pregnancy abortion and clinical pregnancy loss). (3) Three or more times of embryo transfer without clinical pregnancy. (4) Patients with hydrosalpinx. (5) Patients with uncontrolled hyperprolactinemia, hypothyroidism and severe medical or surgical diseases. The institutional ethical board of the First Affiliated Hospital of Sun Yat-sen University approved this retrospective study.

### CT detection and treatments

All patients were required to detect CT before oocyte retrieval or frozen embryo transfer every 6 months in IVF/ICSI cycles. The specimens were cervical swabs detected chlamydial lipopolysaccharide (LPS) by enzyme immunoassay (EIA). Patients tested positive were treated with a 7-day regimen of azithromycin or doxycycline before IVF/ICSI according to the recommendations of the Center for Disease Control and Prevention [15]. Sexual partners of CT-positive patients were treated at the same time. Only when patients retested negative after completing therapy can they enter IVF/ICSI-ET or frozen embryo transfer cycles.

### Ovarian stimulation

Patients received the luteal-phase gonadotrophin-releasing hormone agonist or gonadotrophin antagonist protocols. Since the pituitary was adequately suppressed, or on the 2–3 day of menstruation, ovarian stimulation was induced with human menopausal gonadotrophin and recombinant FSH individualized. When two or more dominant follicles reached 18 mm in diameter, human chorionic gonadotrophin (hCG) was injected and oocytes were collected 36 h after injection.

### Outcome measures

An infertility investigation was performed in all couples. We collected and analyzed data of general characteristics, ovulation induction, embryonic development and pregnancy outcomes. The primary outcome was live-birth rates, the secondary outcomes were the embryo implantation rate, chemical pregnancy rate, ectopic pregnancy rate, abortion rate and clinical pregnancy rate.

In order to investigate whether the interval between CT infection and embryo transfer had any effective on pregnancy outcomes, we further divided the infection group into two subgroups: ≤ 3 months and > 3 months according to the intervals between CT infection and embryo transfer. Data of two subgroups were also compared.

### Statistical analysis

The statistical analyses were performed by SPSS 24.0 software. Pearson’s Chi square test was used to analyze differences between relative frequencies. Student’s t-test and one-way ANOVA were used to compare continuous variables. *P* < 0.05 was considered statistically significant. The multivariate logistic regression model was fitted to analyze the effect of CT infection and the intervals between CT infection and embryo transfer on live birth adjusting for the study variables (baseline characteristics, protocols, and transfer cycles) for live birth rate. Odds ratios or hazard ratios and 95% confidence intervals were calculated. A two-sided a level of 0.05 was considered as statistically significant.

## Results

### Baseline characteristics

**Fig. 1:**
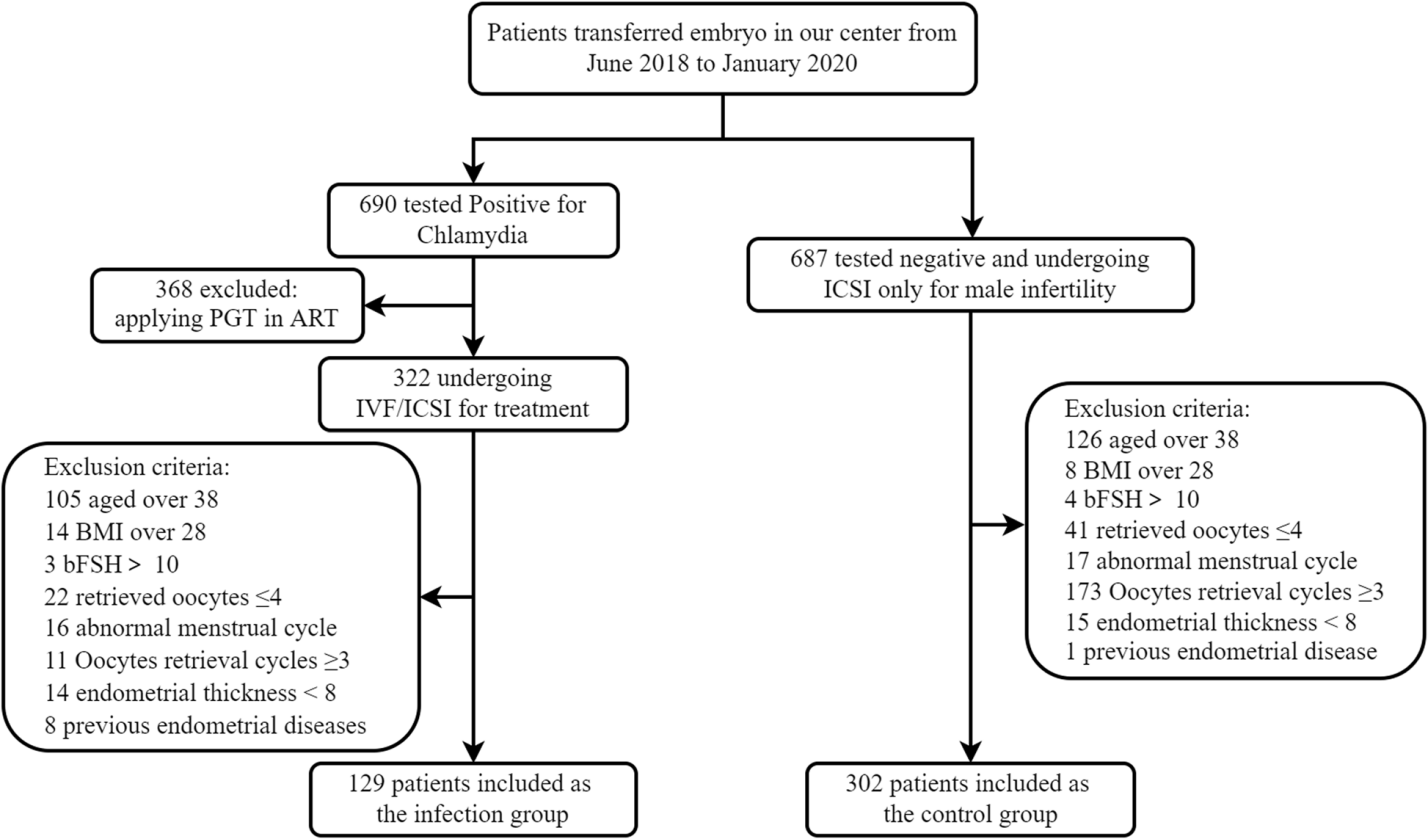
The recruitment process

From June 2018 to January 2020, a total of 690 patients were tested positive for *C. trachomatis* during the inclusion period, of whom 129 couples were eligible and among 687 patients tested CT negative and undergoing ICSI only for male infertility, 302 were enrolled for this analysis (Fig. 1).

**Fig. 2:**
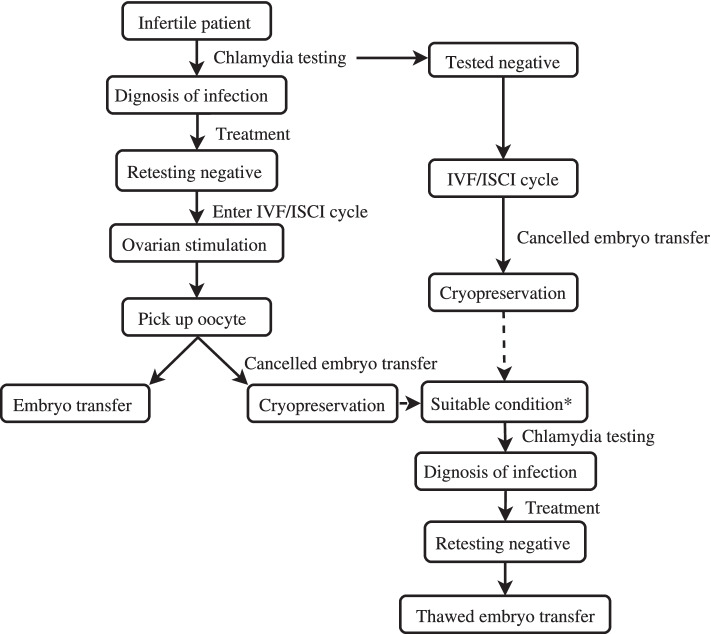
Timeline of Diagnosis and IVC/ICSI Stage of the Infected Group. *Suitable condition: the factors leading to cancelled fresh embryo transfer were overcome, including OHSS, elevated progesterone or CA125 levels, endometrial factors and personal reasons like psychological or financial reason

In the infection group, 88 couples underwent IVF, 37 underwent ICSI and 4 underwent IVF + ICSI. The timeline of diagnosis and IVC/ICSI stage of infected group shown in Fig. 2. The fresh cycle of 74 couples in the infection group and 151 in the control group were cancelled mainly for ovarian hyperstimulation syndrome (OHSS), elevated progesterone or CA125 levels, endometrial factors and personal reasons like psychological or financial reason. Once the factors which lead to cancelled embryo transfer were overcome, couples need to retest CT infection and underwent thawed embryo transfer (TET) after testing negative.

Cycle baseline characteristics were similar between CT positive and negative group, such as age, BMI, infertility duration and basic FSH, while the incidence of secondary infertility and previous ectopic pregnancy rate was higher in CT infection group than that in control group (Table 1).

**Table 1:** Baseline characteristics of the infection and control group

Indexes	Infection group (*n* = 129)	Control group (*n* = 302)	P
Age (years)	30.82 ± 3.80	30.85 ± 3.65	0.939
BMI (kg/m2)	21.19 ± 2.25	21.20 ± 2.34	0.962
Infertility duration (years)	3.40 ± 2.05	3.73 ± 2.56	0.208
Basal FSH (IU/L)	5.35 ± 1.38	5.52 ± 1.40	0.237
Types of infertility (%)			0.028*
Primary infertility	48.8(63/129)	60.3(182/302)	
Secondary infertility	51.2(66/129)	39.7(120/302)	
Previous ectopic pregnancy (%)	16.27(21/129)	1.98(6/302)	< 0.001*

Continuous variables are presented as the mean ± SD

There were no significant differences between two groups concerning initial dose of gonadotropins (Gn), days of Gn used, total dose of Gn, level of serum E_2_ and endometrial thickness on the day of hCG injection, number of retrieved oocytes, blastocyst formation rate and type of embryo transfer, but infection group had lower good-quality embryo rate and embryo transfer numbers, higher blastocyst transfer rate (Table 2).

**Table 2:** Parameters of patients with and without *C. trachomatis* infection

Indexes	Infection group (*n* = 129)	Control group (*n* = 302)	P
Initial dose of Gn (IU)	210.48 ± 58.38	201.44 ± 55.42	0.175
Days of Gn used (IU)	10.69 ± 2.39	10.07 ± 1.73	0.060
Total dose of Gn (IU)	2279.70 ± 961.29	2086.76 ± 800.81	0.054
level of E_2_ on hCG day (ng/ml)	3203.27 ± 1244.91	2986.53 ± 1271.71	0.157
Endometrial thickness (mm)	11.70 ± 2.20	11.79 ± 3.15	0.804
Numbers of oocytes retrieved	15.80 ± 6.96	15.79 ± 6.96	0.990
Good-quality embryo rate (%)	83.3(649/779)	86.5(1438/1662)	0.041*
Blastocyst formation on Day 5 or 6(%)	63.3(584/922)	61.2(1217/1987)	0.282
Transferred embryo stage (%)			0.006*
D3	53.5(69/129)	67.5(204/302)	
D5/6	46.5(60/129)	32.5(98/302)	
Type of embryo transfer (%)			0.161
TET	57.4(74/129)	50(151/302)	
Fresh cycle	42.6(55/129)	50(151/302)	
Number of transferred embryos (%)			0.003*
1	51.9(67/129)	36.4(110/302)	
2	48.1(62/129)	63.6(192/302)	

### Pregnancy outcomes

The implantation rate (28,2% vs 36.2%, *P* = 0.046) was found significantly lower and the PROM rate (7.8% vs 0.7%, *P* = 0.025) was higher in CT infection group comparing with the CT negative. No significant difference was found in the chemical pregnancy rate (48.1%vs 53.0%, *P* = 0.349), abortion rate (21.6% vs 12.7%, *P* = 0.127), clinical pregnancy rate (39.5%vs 47.0%, *P* = 0.152), and ectopic pregnancy rate (2.0% vs 1.3%, *P* = 0.709) between the two groups (Table 3).

**Table 3:** Clinical outcomes of patients with and without *C. trachomatis* infection

Indexes	Infection group (*n* = 129)	Control group (*n* = 302)	P
Implantation rate (%)	28.2(55/195)	36.2(177/489)	0.046*
Chemical pregnancy rate (%)	48.1(62/129)	53.0(160/302)	0.349
Clinical pregnancy rate (%)	39.5(51/129)	47.0(142/302)	0.152
Ectopic pregnancy rate (%)	2.0(1/51)	1.3(2/160)	0.709
Gestational diabetes mellitus (%)	0.0(0/51)	9.9(14/142)	0.044*
Hypertensive disorders of pregnancy (%)	0.0(0/51)	4.9(7/142)	0.239
Hyperthyroidism in pregnancy (%)	0.0(0/51)	0.7(1/142)	1.000
Placenta previa (%)	0.0(0/51)	0.7(1/142)	1.000
Premature rupture of membranes (%)	7.8(4/51)	0.7 (1/142)	0.025*
Oligohydramnios (%)	0.0(0/51)	0.7(1/142)	1.000
Abortion rate (%)	21.6(11/51)	12.7(18/142)	0.127
Live birth rate (%)	30.2(39/129)	40.1(121/302)	0.053
Gestational age (w)	38.3 ± 1.9	38.1 ± 2.1	0.670
Premature birth rate (%)	20.5(8/39)	21.7(26/120)	0.879
Pulmonary embolism (%)	0.0(0/39)	0.8 (1/120)	1.000

During pregnancy, seven patients developed hypertensive disorders of pregnancy, one developed hyperthyroidism in pregnancy, one suffered with placenta previa and one had low amniotic fluid in the control group, which are not statistically significant comparing to the infection group. The rate of gestational diabetes mellitus is slightly higher in the control group than the infection group.

As for conditions of birth and delivery, the rates of abortion, live birth and premature birth and the average birth weight of newborn are similar in both groups. One patient in the control group was diagnosed pulmonary embolism in the postpartum period. There was one neonate with cryptorchidism, one with talipes equinovarus and one with gastroschisis in the control group and one in the infection group was born with congenital heart diseases.

Previous studies demonstrated relative factors which can become potential confounders in this study [16, 17]. The univariate analysis showed live birth rate was associated with age, BMI, infertility duration, initial dose of Gn, total dose of Gn, level of E_2_, approach of fertilization and whether CT infection or not *(P < 0.1)* (Table 4).

**Table 4:** Univariate analysis of live birth rate

Indexes	OR (95%CI)	P
Age (years)	0.926(0.880,0.975)	0.003^*^
BMI (kg/m2)	1.050(0.996,1.106)	0.069^*^
Infertility duration (years)	0.922(0.847,1.004)	0.061^*^
Basal FSH (IU/L)	0.957 (0.852,1.075)	0.461
Types of infertility (%)	0.977(0.659,1.449)	0.907
Initial dose of Gn (IU)	0.994(0.991,0.998)	0.001^*^
Days of Gn used (IU)	0.922(0.829,1.025)	0.135
Total dose of Gn (IU)	1.000(0.999,1.000)	0.002^*^
level of E2 on hCG day (ng/ml)	1.000(1.000,1.000)	0.075^*^
Endometrial thickness (mm)	0.985(0.919,1.056)	0.676
Transferred embryo stage (%)	0.781(0.523,1.167)	0.228
Type of embryo transfer (%)	0.968(0.651,1.437)	0.870
Number of transferred embryos (%)	0.924(0.624,1.367)	0.691
Approach of fertilization	0.521(0.310,0.878)	0.014^*^
Previous CT infection	0.648(0.417,1.007)	0.054^*^

A multivariable model was performed to adjust for these significant factors and type of embryo transfer [17–19]. There was no significant difference found between control and infection groups *(p > 0.05)* (Table 5).

**Table 5:** Multivariable logistic regression of the live birth rate

Indexes	B	SE	Waldχ2	P	OR (95%)
Age (years)	−0.044	0.030	2.103	0.147	0.957(0.901,1.016)
BMI (kg/m2)	0.053	0.031	2.912	0.088	1.054(0.992,1.119)
Infertility duration (years)	−0.065	0.046	2.011	0.156	0.937(0.856,1.025)
Initial dose of Gn (IU)	−0.002	0.003	0.585	0.444	0.998(0.992,1.003)
Total dose of Gn (IU)	0.000	0.000	1.015	0.314	1.000(0.999,1.000)
level of E2 on hCG day (ng/ml)	0.000	0.000	0.149	0.700	1.000(1.000,1.000)
Approach of fertilization	−0.605	0.417	2.108	0.146	0.546(0.241,1.236)
Type of embryo transfer	0.047	0.226	0.043	0.836	1.048(0.673,1.631)
Previous CT infection	0.010	0.373	0.001	0.979	1.010(0.486,2.096)

### The pregnancy outcomes of different intervals between infection and embryo transfer

According to previous study (Patton et al.,1994), the detection of antibody against CT may associate with the intervals from the end of treatment. Based on the findings above and the clinical experience, we further subdivided the infection group into two subgroups for ≤3 months and > 3 months, according to the different intervals between CT infection and embryo transfer. All patients in CT positive group were treated with a 7-days regimen and retested CT negative before transferring embryos. The characteristics and outcomes of the two subgroups were also compared (Table 6).

**Table 6:** Characteristics and outcomes after IVF/ICSI-ET in the infected group

Indexes	≤3 months (*n* = 60)	> 3 months (*n* = 69)	P
Age (years)	31.35 ± 3.89	30.95 ± 3.82	0.263
BMI (kg/m2)	21.32 ± 2.31	21.01 ± 2.39	0.455
Basal FSH (U/L)	5.48 ± 1.44	5.26 ± 1.34	0.385
Types of infertility (%)			0.061
Primary infertility	40 (24/60)	56.5 (39/69)	
Secondary infertility	60(36/60)	43.5 (30/69)	
Endometrial thickness (mm)	11.71 ± 2.21	11.60 ± 2.36	0.789
Approach of Fertilization (%)			0.979
IVF	68.3 (41/60)	68.1 (47/69)	
ICSI/IVF + ICSI	31.7 (19/60)	31.9 (22/69)	
Type of embryo transfer (%)			0.053
TET	48.3 (29/60)	65.2 (45/69)	
Fresh cycle	51.7 (31/60)	34.8 (24/69)	
Number of transferred embryos (%)			0.767
1	53.3 (32/60)	50.7 (35/69)	
2	46.7 (28/60)	49.3 (34/69)	
Transferred embryo stage (%)			0.500
D3	56.7 (34/60)	50.7 (35/69)	
D5/6	43.3 (26/60)	49.3 (34/69)	
Good-quality embryo rate (%)	86.7 (284/328)	80.9 (365/451)	0.034*
Blastocyst formation rate (%)	59.9 (240/400)	65.9 (344/522)	0.064
Chemical pregnancy rate (%)	38.3 (23/60)	50.7 (35/69)	0.158
Clinical pregnancy rate (%)	36.7(22/60)	42.0(29/69)	0.534
Premature rupture of membranes (%)	4.5(1/22)	13.6(3/22)	0.600
Abortion rate (%)	22.7(5/22)	20.7(6/29)	1.000
Live birth rate (%)	28.3(17/60)	31.9(22/69)	0.661
Gestational age(w)	37.4 ± 3.5	38.2 ± 2.4	0.417
Premature birth rate (%)	13.6(3/22)	17.9(5/28)	0.988
Birth weight (g)	3082.9	2844.8	0.271
Macrosomia (%)	4.5 (1/22)	3.6(1/28)	1.000
Normal birth weight infant (%)	81.8(18/22)	75.0(21/28)	0.815
Low birth weight infant (%)	13.6(3/22)	10.7(3/28)	1.000
Very low birth weight infant (%)	0(0/22)	10.7(3/28)	0.325
Congenital malformation (%)	4.5(1/22)	0(0/28)	0.440

The baseline characteristics were similar and comparable. Patients with interval > 3 months showed slightly lower good-quality embryo rate than those ≤3 months. Except for this, there were no significant differences in pregnancy outcomes between the subgroups.

The multivariate logistic regression showed no significant difference in the clinical outcomes when analyzing with correction for the significant factors, type of embryo transfer and the time interval ≤ 3 months or not *(P > 0.05)* (Table 7).

**Table 7:** Multivariable logistic regression of the live birth rate in the infection group

Indexes	B	SE	Waldχ2	P	OR (95%)
Age (years)	−0.066	0.055	1.401	0.237	0.936(0.840,1.044)
BMI (kg/m2)	0.058	0.040	2.163	0.141	1.060(0.981,1.145)
Infertility duration (years)	−0.044	0.104	0.180	0.671	0.957(0.781,1.172)
Initial dose of Gn (IU)	−0.004	0.005	0.751	0.386	0.996(0.986,1.005)
Total dose of Gn (IU)	0.000	0.000	0.048	0.826	1.000(0.999,1.001)
level of E2 on hCG day (ng/ml)	0.000	0.000	0.329	0.566	1.000(0.999,1.000)
Approach of fertilization	−5.27	0.454	1.350	0.245	0.590(0.243,1.436)
Type of embryo transfer	0.874	0.686	1.624	0.203	2.398(0.625,9.202)
Previous CT infection	−0.201	0.451	0.198	0.656	0.818(0.338,1.979)

## Discussion

As we all know, the ICSI cycles have similar pregnancy outcomes to the IVF cycles [20]. In this study, women who underwent ICSI-ET treatment without fallopian tube factors were included in our control group, so as to minimize the impact of fallopian tube problems probably caused by previous CT infection on the outcome of the control group. The risk of major birth defects after ICSI were not found in the control group [21]. The infection and control groups were comparable regarding demographic data, infertility duration, duration of stimulation, duration of treatment, E_2_ level and endometrial thickness on the day of HCG administration. There was no statistically significant difference observed between the two groups regarding the mean number of oocytes retrieved per patient and blastocyst formation rate, but the mean number of embryos transferred per patient and good quality embryos rate were significantly lower while the rate of premature rupture of membranes was higher in infection group than in control group.

It had suggested that adverse reproductive sequelae of ectopic pregnancy can be contributed to the antibody response to the chlamydial heat shock protein 60 (CHSP 60) [4, 5, 22]. In our infection group, infertile women combined with tubal factors had higher incidence of previous ectopic pregnancy than the control group without tubal factors. However, after IVF/ICSI-ET treatment, CT infection did not increase the incidence of ectopic pregnancy significantly. We speculated the possible reason was that previous studies on the impact of CT on natural conception did not address whether CT was prescriptively treated and turned negative before pregnancy, and some were even found to be CT positive during pregnancy. While in this study, patients tested positive started IVF/ICSI-ET cycles only after prescriptive therapy and the cervical swab resulted negative for Chlamydia.

The impacts of CT on the pregnancy outcomes in assisted reproductive technology are still controversial. Several researchers demonstrated negative effects of previous chlamydial infection. According to Witkin et al. [10], the inflammatory reaction in the uterus, resulting from the prevalence of CHSP 60 antibodies, can impair embryo implantation. Pacchiarotti et al. [9] found the presence of anti-chlamydia antibodies can lead to fewer mature oocytes end poor IVF outcomes. On the contrary, some studies showed no effect of previous CT infection on pregnancy outcomes in IVF/ICSI [11–13, 23]. Claman et.al [24] found no differences in pregnancy rates or outcomes in patients with and without serologic evidence of previous CT infection. Nevertheless, Serum anti-chlamydial antibodies do not correlate as well directly with cervical infection. When suffering from CT infection, the expression and distribution of antibodies are not synchronous [7]. Therefore, the misclassification of chlamydia stages may account for different conclusions of the studies on CT infection.

A global systematic review [25] demonstrates associations between chlamydia and several adverse pregnancy outcomes. But there are some limitations due to the substantial heterogeneity across studies and the confounding variables such as medical history. The possible impacts of the related factors on the results cannot be explained when quantifying the correlation. In addition, differences of study population, sample size, treatment and random error may also have certain impact on the result. Our results of multivariable regression analysis showed that CT infection was not significantly associated with lower chemical pregnancy rate, clinical pregnancy rate, abortion rate and live birth rate, which confirmed that previous CT infection is not a risk factor of adverse pregnant outcomes of IVF/ICSI. The rate of premature rupture of membranes was higher in infection group than in control group. The persistent subclinical chlamydial infection and the abnormal immune induced by previous CT infection may increase the concentration of polymorphonuclear leukocytes which is capable of altering the local tissue pH and thus perturbing lysosomal membranes in the adjacent chorioamnion and histological chorioamnionitis. The effect could lead to weaken membranes and hence membrane rupture [26, 27].

According to previous study [28], the detection of antibody against CT may associate with the intervals from the end of treatment. Based on the findings above and the clinical experience, we further subdivided the infection group into two subgroups for ≤3 months and > 3 months. There was no differences between the subgroups, showing that the group > 3 months interval between CT infection diagnosis and embryo transfer have similar outcomes in the IVF/ICSI to those ≤3 months. According to Hoenderboom et.al [14]. Women with previous CT infection have similar overall pregnancy rates as those tested negative but took longer to get pregnant possibly because of intratubal microdamage. Therefore, we suggested that the infection of CT without hydrosalpinx will not impair the ability of embryos implantation and development after their transfer to the uterus. However, the PROM rate showed higher in the infection group than the control group while no different was found between the subgroups. It suggested that longer time interval would not reduce the impact of CT infection on the risk of PROM.

In this study, we formulated strict inclusion criteria, exclusion criteria and perform a multivariable logistic regression, which is conducive to eliminate the possible effects of general characteristics in the transplantation cycles. Compared with previous studies, we greatly controlled the possible confounders between the infection group and the control group. Subgroup stratification was performed in the infected group to analyze whether the time intervals between infection and embryo transfer had any effect on pregnancy outcomes. The results showed that prolonged interval did not increase implantation rate, clinical pregnancy rate, live birth rate, and did not reduce miscarriage rate when the interval > 3 months verse ≤3 months. Our results suggest that there is no need to delay embryo transfer after cure of CT infection during the IVF/ICSI cycle.

## Conclusion

This study demonstrates that cured CT infection without hydrosalpinx will not impair embryo implantation, clinical pregnancy and live birth but lead women at high risk of PROM during IVF/ICSI. Although CT infection does not significantly affect the live birth rate, it can result in an increased incidence of PROM, while extended interval between infection and embryo transfer will not decrease the risk of adverse outcomes, which may provide a good warning for clinical practice. There are still many deficiencies due to the limited sample size, testing technology and other reasons. We have not analyzed the long-term effects of previous CT infection to the infants. The effects of longer or shorter time intervals on pregnancy outcomes are unknown yet. Further study is warranted to confirm our proposal.

## Data Availability

The datasets analyzed during the current study are not publicly available given the privacy of patient data and we plan to further analyze the data to dig up much more clinic information. But they are available from the corresponding author on reasonable request.

## Abbreviations

IVF: In-Vitro Fertilization
ICSI: Intracytoplasmic sperm injection
Gn: Gonadotropins
CT: *Chlamydia trachomatis*
HSP 60: Heat shock protein 60
CHSP 60: Chlamydial heat shock protein 60
